# Patterns of motivators and barriers to heart health behaviors among adults with behavior-modifiable cardiovascular risk factors: A population-based survey in Singapore

**DOI:** 10.1371/journal.pone.0262752

**Published:** 2022-01-20

**Authors:** Zijuan Huang, Jien Sze Ho, Qai Ven Yap, Yiong Huak Chan, Swee Yaw Tan, Natalie Koh Si Ya, Lip Ping Low, Huay Cheem Tan, Woon Puay Koh, Terrance Siang Jin Chua, Sungwon Yoon

**Affiliations:** 1 National Heart Centre Singapore, Singapore, Singapore; 2 Department of Biostatistics, Yong Loo Lin School of Medicine, Singapore, Singapore; 3 Low Cardiology Clinic, Mount Elizabeth Medical Centre, Singapore, Singapore; 4 Department of Cardiology, National University Heart Centre, Singapore, Singapore; 5 Healthy Longevity Translational Research Programme, Yong Loo Lin School of Medicine, National University of Singapore, Singapore, Singapore; 6 Singapore Institute for Clinical Sciences, Agency for Science Technology and Research (A*STAR), Singapore, Singapore; 7 Health Services and Systems Research, Duke-NUS Medical School, Singapore, Singapore; 8 Centre for Population Health Research and Implementation (CPHRI), SingHealth Regional Health System, SingHealth, Singapore, Singapore; Kuwait University Faculty of Dentistry, KUWAIT

## Abstract

**Objectives:**

Motivators and barriers are pivotal factors in the adoption of health behaviors. This study aims to identify patterns of the motivators and barriers influencing heart health behaviors among multi-ethnic Asian adults with behavior-modifiable risk factors for heart disease, namely obesity, physical inactivity and smoking.

**Methods:**

A population-based survey of 1,000 participants was conducted in Singapore. Participants were assessed for behavior-modifiable risk factors and asked about motivators and barriers to heart health behaviors. Exploratory and confirmatory factor analyses were conducted to identify factors underlying motivator and barrier question items. Logistic regression was conducted to examine the associations of motivator and barrier factors with sociodemographic characteristics.

**Results:**

The twenty-five motivator and barrier items were classified into three (outcome expectations, external cues and significant others including family and friends) and four (external circumstances, limited self-efficacy and competence, lack of perceived susceptibility, benefits and intentions and perceived lack of physical capability) factors respectively. Among participants with behavior-modifiable risk factors, those with lower education were more likely to be low in motivation factor of “outcome expectations” and “external cues”. The well-educated were more likely to be high in the barrier factor of “lack of perceived susceptibility, benefits and intention” and were less likely to have the motivation factor of “significant others (family or friends)”. Those aged 60–75 years had low motivations and high barriers compared to their younger counterparts. Older age was more likely to be low in motivation factor of “outcome expectations” and “external cues” and high in barrier factor of “limited self-efficacy and competence” and “perceived lack of physical capability”.

**Conclusions:**

Findings underscore the importance of a targeted intervention and communication strategy addressing specific motivation and barrier factors in different population segments with modifiable risk factors.

## Introduction

Cardiovascular disease (CVD) is largely preventable by addressing modifiable risk factors and adopting heart health behaviors [1–3]. Despite positive health benefits of heart health behaviors, a significant proportion of adults across different populations still lack adequate heart healthy behaviors [4–6]. It is also increasingly evident that knowledge alone is insufficient to change behavior with multiple studies demonstrating a significant disconnect between knowledge and behavior [7, 8].

Evidence demonstrates that ability or inability to initiate and sustain heart health behaviors can be explained by motivators and barriers [9, 10]. Without understanding factors that enable or impede an individual’s ability to change health behaviors, heart disease prevention efforts may be unlikely to achieve their intended impact. This is particularly important for those whose CVD risk factors are modifiable, but do not undertake healthy behaviors.

Existing literature suggests that various factors influence the engagement in heart healthy behaviors among adults. They include support from family and friends, beliefs about the causes of illness, lack of time and access issues such as transport and financial costs [11–13]. However, few studies have examined Asian adults at the population level. In addition, existing population surveys tended to focus on population in general as opposed to a population segment who have modifiable CVD risks through behavior changes. As populations may differ in social, cultural and environmental aspects, it is important to conduct population-specific and segment-based assessment of factors that influence heart healthy behaviors. Since the motivators and barriers are often distinct and heterogenous across individuals, categorizing them into several broad components would allow for understanding the patterns of motivators and barriers thereby guiding future efforts for health promotion.

To this end, we conducted a population survey to examine the factors that motivate and hinder uptake of heart health behavior in our Asian population. These motivators and barriers have previously been surveyed in the Western population [14]. The present study examined patterns of motivators and barriers among adult segments who have behavior-modifiable risk factors for heart disease—obesity [15], physical inactivity [16] and smoking [17, 18]. We also sought to examine the association of the patterns of motivators and barriers and sociodemographic characteristics among these three different risk groups.

## Methods

### Sample

This is a population-based survey conducted in Singapore. Eligibility criteria included Singapore citizens or permanent residents of both genders aged 21 to 75 years old. Stratified cluster random sampling was used. A list of addresses was obtained from the Singapore Department of Statistics. The residential areas were divided into 26 subzones, and selection was stratified by subzone then by housing type (public housing, landed property, condominium, shop house). Within each housing type, blocks and units were randomly selected. There was a quota for gender and age group to ensure that participants’ demographics were matched with the national distribution. We oversampled certain ethnic groups to obtain a nearly equal proportion of three major ethnic groups in Singapore (i.e. Chinese, Malays and Indians). The race distribution in Singapore is approximately 70% Chinese, 20% Malay and 10% Indian.

### Instrument

We used a structured questionnaire to collect information on socio-demographics, behavior-modifiable risk factors and motivators and barriers to the uptake of heart healthy behaviors.

#### Sociodemographic variables

We collected age, gender, ethnicity, education and employment status. Education was classified into “primary or below” (primary school or lower level of education), “university or above” (university level education or above) and “intermediate” (education level between “primary or below” and “university or above”). Employment status was classified into “working”‘ (full time employed, part time employed, self-employed, employers), “retired/homemakers” (retirees and homemakers), “others” (national servicemen, students, unemployed).

#### Behavior-modifiable cardiovascular risk factors: Physical inactivity, smoking and obesity

Participants were asked about their physical activities in the past month with a series of questions adopted from the Behavioral Risk Factor Surveillance System (BRFSS) [19]. Physical activities cited were matched to their respective metabolic equivalent of task [20] (MET) values based on the BRFSS activity table and classified into low, moderate and vigorous intensity exercise. Time spent in activities per week was computed based on the answers given on time spent on each activity. The MET data was then dichotomized into “adequate exercise” (which met heart healthy activity recommendations of 150 min per week of moderate intensity aerobic exercise or 75 min per week of vigorous aerobic exercise or equivalent combinations) and “inadequate exercise” (which did not meet heart healthy activity recommendations). Physical inactivity was defined as not achieving the recommendations (i.e., inadequate exercise). Participants were also asked about smoking status [21], whether they had smoked 100 cigarettes in their lifetime with options being ‘yes’, ‘no’, ‘don’t know’. If they answered ‘yes’, they were classified as “ever smokers”. Ever smokers were then asked whether they currently were smoking ‘every day’, ‘some days’ or ‘not at all’. ‘Not at all’ response was classified as “not currently smoking”. ‘Some days’ and ‘every day’ responses were classified as “current smokers”. Lastly, participants’ self-reported height (cm) and weight (kg) were collected, body mass index [22] (BMI) was then calculated from these values. BMI categories were defined using national cut off points, that is BMI ≥ 27.5 for obese, 23 ≤ BMI < 27.5 for overweight, BMI < 23 for normal or low BMI.

#### Motivators and barriers

Items on motivators and barriers to lifestyle changes were developed based on a similar study conducted in the United States [14]. The items on motivators and barriers to lifestyle changes presented a high internal consistency, both with Cronbach’s alpha 0.904. Participants were presented with examples of heart healthy actions (e.g., quit smoking, have regular physical exercise, lose weight, reduce cholesterol intake, reduce stress, reduce sodium intake, maintain a healthy blood pressure, moderate alcohol consumption, increase fruits/vegetable intake, get adequate sleep, reduce sugar intake, visit a doctor to get regular tests for heart disease prevention). Participants were asked if they had taken some of these actions, how much was driven by certain motivators listed (e.g. because I live longer, I was encouraged by family members); and if they had not taken these actions, how much was due to certain barriers listed (e.g. I don’t think I am at risk of heart disease, I have no time to change my lifestyle) on a 4-point Likert scale of agreement (1 = strongly agree/2 = agree/3 = disagree/4 = strongly disagree). Each response to motivator items was reversely coded. Total score was calculated and dichotomized into low motivation (<mean score-1SD) and high motivation (>= mean score-1SD). Each response to barrier items was summed and dichotomized into high barrier (<mean score-1SD) and low barrier (>= mean score-1SD).

### Procedure

The questionnaire was translated into English, Chinese, Malay and Tamil. A forwards-backwards translation was performed to ensure that the questionnaires were conceptually equivalent. An initial pilot survey was tested in 10 respondents and revisions were made based on feedback provided. Trained interviewers went door-to-door to administer the survey. The survey was administered in one of the four national languages (English, Mandarin, Malay or Tamil), and was carried out from April 2018 to May 2018. As the survey was anonymous, verbal consent was obtained by the interviewer before the survey was conducted. This study was approved by National University of Singapore’s Institutional Review Board (S-17-256E).

### Sample size

From prior estimates of individuals with suboptimal heart health behavior such as lack of exercise in Singapore, about one third did not have adequate exercise [23]. Planning for a 1-month recruitment period, 1,000 participants were invited. This would provide a precision of 3% on a postulated prevalence of 30% (highest) for any one of the three behavior-modifiable risk factors to be studied.

### Statistical analysis

Descriptive statistics for socio-demographic numerical variables were presented as mean (SD) and n (%) for categorical variables. Cronbach alphas were presented to show the reliability of the question items on motivators and barriers. Factor analysis was conducted to classify motivator and barrier question items into groups. This was done by first conducting exploratory factor analysis, to assess the underlying factor structure of motivator and barrier question items. This was validated by confirmatory factor analysis (CFA). The factors that the motivators and barrier questions were grouped under were then defined. The associations of motivators, barriers and socio-demographics in adult population segments with behavior-modifiable risk factors (physical inactivity, smoking, obesity) were determined using weighted (by race) logistic regression. Variables with p-value<0.2 from univariate logistic regression were entered into multivariate logistic regression models. Statistical analysis was performed using STATA version 16 with statistical significance set at 2-sided p < 0.05.

## Results

### Sociodemographic characteristics

Of 1,000 participants who were interviewed, 357(35.7%), 321(32.1%), 322(32.2%) were Chinese, Malays and Indian, respectively. All were Singaporeans or permanent residents in Singapore. More than two thirds of participants had an intermediate level of educational attainment and about two thirds were in the workforce (Table 1). There was no missing data.

**Table 1:** Demographics of survey respondents (n = 1,000).

Variable	Category	Whole Cohort	Chinese	Malay	Indian
Age group	20–39 years old	385	123 (34.5)	138 (43)	124 (38.5)
	40–59 years old	395	137 (38.4)	124 (38.6)	134 (41.6)
	60–75 years old	220	97 (27.2)	59 (18.4)	64 (19.9)
Gender	Female	521	186 (52.1)	168 (52.3)	167 (51.9)
	Male	479	171 (47.9)	153 (47.7)	155 (48.1)
Education level	Low education (primary school or less)	194	65 (18.2)	70 (21.8)	59 (18.3)
	Intermediate education	679	216 (60.5)	234 (72.9)	229 (71.1)
	High education (university or above)	127	76 (21.3)	17 (5.3)	34 (10.6)
Employment	Employed full time	551	197 (35.75)	177 (32.12)	177 (32.12)
	Employed part time	78	22 (28.21)	28 (35.90)	28 (35.90)
	Employer/self employed	36	19(52.78)	8(22.22)	9(25.00)
	Unemployed looking for work	13	1(7.69)	7(53.85)	5(38.46)
	Not working not looking for work	39	11(28.21)	10(25.64)	18(46.15)
	Retired	94	42(44.68)	27(28.72)	25(26.60)
	Homemaker	141	49(34.75)	45(31.91)	47(33.33)
	Full time National Service	10	4(40.00)	6(60.00)	0(0)
	Full time student	38	12 (31.58)	13 (34.21)	13 (34.21)
BMI	Low risk	418	173 (48.46)	131 (40.81)	114 (35.4)
	Moderate risk	417	143 (40.06)	126 (39.25)	148 (45.96)
	High risk	165	41 (11.48)	64 (19.94)	60 (18.63)

Note: Values are in n (%).

### Exploratory factor analysis (EFA)

EFA classified motivator question items into three factors and barrier question items into four factors. The items were assigned to the factor on which it had the highest factor loading. The three factors of motivator question items were named *outcome expectations*, *external cues* and *significant others (family or friends)*. While the four factors of barrier question items were named e*xternal circumstances*, *limited self-efficacy and competence*, *lack of perceived susceptibility*, *benefits and intentions* and *perceived lack of physical capability* (Table 2).

**Table 2:** Exploratory and confirmatory factor analysis.

Factors	Items	Factor loadings	CFI
**Motivators**			
Outcome expectations	Live longer	0.727	0.935
	Feel better	0.830	
	Improve my health	0.806	
	Avoid taking medications	0.784	
	Do it for my family	0.759	
External cues	Doctor/nurse	0.594	0.989
	Information on heart disease in the media (newspapers and TV)	0.857	
	Community health events	0.882	
	Death and illness of family members from heart disease	0.731	
Significant others (family or friends)	Family members	0.800	1.000
	Friends	0.792	
**Barriers**			
External circumstances	I have too many things on my mind/distracted/depressed/preoccupied/stressed to change my lifestyle.	0.525	0.975
	I don’t have the money to change my lifestyle.	0.757	
	I have no time to change my lifestyle.	0.800	
	I feel the changes required are too difficult.	0.769	
	I don’t know what I should do.	0.669	
	There is too much confusion in the media about what to do to change my lifestyle.	0.488	
Limited self-efficacy and competence	I have tried but failed to change my lifestyle.	0.768	1.000
	I’m not confident that I can change my lifestyle.	0.661	
	My doctor doesn’t explain clearly what I should do.	0.736	
Lack of perceived susceptibility, benefits and intentions	I don’t think I am at risk of heart disease.	0.851	1.000
	I don’t want to change my lifestyle.	0.856	
	Changing my behavior will not reduce my risk of heart disease.	0.732	
Perceived lack of physical capability	I am too ill to change my lifestyle.	0.756	1.000
	I am too old to change my lifestyle.	0.876	

### Confirmatory factor analysis (CFA)

CFA confirmed the factor structure identified by EFA with high goodness of fit. (All CFI>0.9) (Table 2).

### The physically inactive

#### Total scores of motivators and barriers in the physically inactive

Among physically inactive individuals, lower education (primary level education or below) was significantly associated with having low motivations (adjusted OR 2.4, 95% CI 1.4–4.2, p = 0.001) as compared to intermediate level education. Those with intermediate level education and university level education or above were significantly more likely to have high barriers as compared to those with primary level education or below (adjusted OR 2.4, 95% CI 1.1–4.9, p = 0.022 and adjusted OR 5.1, 95% CI 2.1–12.5, p<0.001, respectively) (Table 3).

**Table 3:** Motivators and barriers among the physically inactive: Overall score and factors.

**Motivators (total score)**						
**Variable**	**Motivated**	**Very Unmotivated**	**Unadjusted**		**Adjusted**	
			**OR (95% CI)**	**P-value**	**OR (95% CI)**	**P- value**
**Race**						
Chinese	361(80.0%)	90(20.0%)	1.6(0.9–2.8)	0.091	1.7(1.0–3.0)	0.070
Indian	56(80.0%)	14(20.0%)	1.6(0.7–3.4)		1.6(0.7–3.7)	
Malay	116(86.6%)	18(13.4%)	1.0	0.253	1.0	0.217
**Age group**						
20–39	183(81.0%)	43(19.0%)	1.2(0.8–2.0)	0.388	1.5(0.9–2.4)	0.114
60–75	121(77.1%)	36(22.9%)	1.6(1.0–2.6)		1.2(0.6–2.2)	
40–59	228(84.1%)	43(15.9%)	1.0	0.068	1.0	0.574
**Education**						
Primary or below	106(71.6%)	42(28.4%)	2.3(1.4–3.6)	<0.001	2.4(1.4–4.2)	0.001
University or above	85(80.2%)	21(19.8%)	1.4(0.8–2.4)	0.256	1.4(0.8–2.6)	0.213
Intermediate	342(85.1%)	60(14.9%)	1.0		1.0	
**Employment status**						
Others	39(70.9%)	16(29.1%)	2.1(1.1–4.0)	0.018	2.1(1.1–4.1)	0.035
Retired/homemakers	116(79.5%)	30(20.5%)	1.3(0.8–2.1)	0.275	1.0(0.5–1.9)	0.961
Working	378(83.4%)	75(16.6%)	1.0		1.0	
**Motivator factors**						
**Variable**	**High score**	**Very low score**	**Unadjusted**		**Adjusted**	
			**OR (95% CI)**	**P-value**	**OR (95% CI)**	**P- value**
**Outcome expectations**						
**Age group**						
20–39	156(69.0%)	70(31.0%)	1.2(0.8–1.7)	0.424	1.2(0.8–1.8)	0.361
60–75	97(61.8%)	60(38.2%)	1.6(1.1–2.5)	0.023	1.569(1.004–2.453)	0.048
40–59	196(72.3%)	75(27.7%)	1.0		1.0	
**Education**						
Primary or below	99(67.3%)	48(32.7%)	1.9(1.1–3.5)	0.028	1.7(0.9–3.2)	0.129
Intermediate	266(66.3%)	135(33.7%)	2.0(1.2–3.4)	0.007	1.9(1.1–3.2)	0.018
University or above	85(80.2%)	21(19.8%)	1.0		1.0	
**Gender**						
Male	219(66.0%)	113(34.0%)	1.3(0.9–1.8)	0.128	1.2(0.9–1.7)	0.279
Female	231(71.5%)	92(28.5%)	1.0		1.0	
**Currently smoking**						
Yes	106(62.4%)	64(37.6%)	1.5(1.0–2.1)	0.045	1.3(0.9–2.0)	0.132
No	344(70.9%)	141(29.1%)	1.0		1.0	
**External cues**						
**Education**						
Primary or below	98(66.2%)	50(33.8%)	1.9(1.2–2.8)	0.004	2.0(1.2–3.2)	0.004
University or above	75(70.8%)	31(29.2%)	1.5(0.9–2.4)	0.094	1.6(1.0–2.6)	0.057
Intermediate	315(78.6%)	86(21.4%)	1.0		1.0	
**Employment status**						
Working	343(75.7%)	110(24.3%)	0.97(0.63–1.49)	0.871	1.2(0.7–2.0)	0.517
Others	34(61.8%)	21(38.2%)	1.8(0.9–3.5)	0.080	2.2(1.1–4.4)	0.028
Retired/homemakers	110(75.3%)	36(24.7%)	1.0		1.0	
**Currently smoking**						
Yes	115(67.6%)	55(32.4%)	1.6(1.1–2.4)	0.015	1.6(1.1–2.4)	0.019
No	373(77.1%)	111(22.9%)	1.0		1.0	
**Significant others (family or friends)**						
**BMI**						
Overweight	262(93.6%)	18(6.4%)	1.7(0.8–3.6)	0.202	1.7(0.8–3.8)	0.173
Obese	92(91.1%)	9(8.9%)	2.3(0.9–5.8)	0.077	2.5(0.9–6.6)	0.066
Low or normal	263(96.0%)	11(4.0%)	1.0		1.0	
**Education**						
Primary or below	136(92.5%)	11(7.5%)	2.3(1.0–5.1)	0.043	2.1(0.9–5.3)	0.095
University or above	94(88.7%)	12(11.3%)	3.4(1.5–7.7)	0.003	4.5(1.9–10.7)	0.001
Intermediate	387(96.5%)	14(3.5%)	1.0		1.0	
**Employment status**						
Others	47(85.5%)	8(14.5%)	3.4(1.4–8.1)	0.005	4.3(1.7–11.0)	0.002
Retired/homemakers	139(95.2%)	7(4.8%)	1.0(0.5–2.5)	0.887	1.0(0.4–2.8)	0.973
Working	431(95.1%)	22(4.9%)	1.0		1.0	
**Barriers (total score)**						
**Variable**	**Low**	**Very high**	**Unadjusted**		**Adjusted**	
			**OR (95% CI)**	**P-value**	**OR (95% CI)**	**P- value**
**BMI**						
Overweight	250(89.3%)	30(10.7%)	1.1(0.6–1.9)	0.775	1.1(0.6–2.0)	0.737
Obese	82(81.2%)	19(18.8%)	2.0(1.1–3.8)	0.033	2.3(1.2–4.5)	0.013
Low or normal	246(89.8%)	28(10.2%)	1.0		1.0	
**Education**						
Intermediate	356(88.8%)	45(11.2%)	1.4(0.7–2.7)	0.342	2.4(1.1–4.9)	0.022
University or above	87(82.1%)	19(17.9%)	2.3(1.1–5.0)	0.029	5.1(2.1–12.5)	<0.001
Primary or below	135(91.2%)	13(8.8%)	1.0		1.0	
**Employment status**						
Others	49(89.1%)	6(10.9%)	1.1(0.5–2.7)	0.784	1.4(0.6–3.4)	0.467
Retired/homemakers	123(84.2%)	23(15.8%)	1.6(0.9–2.7)	0.087	2.4(1.2–4.6)	0.010
Working	406(89.6%)	47(10.4%)	1.0		1.0	
**Gender**						
Female	279(86.4%)	44(13.6%)	1.5(0.9–2.4)	0.124	1.3(0.8–2.3)	0.289
Male	299(90.1%)	33(9.9%)	1.0		1.0	
**Barrier factors**						
**Variable**	**High score**	**Very low score**	**Unadjusted**		**Adjusted**	
			**OR (95% CI)**	**P-value**	**OR (95% CI)**	**P- value**
**External circumstances**						
**BMI**						
Overweight	224(80.0%)	56(20.0%)	1.8(1.1–2.9)	0.012	1.9(1.2–3.0)	0.010
Obese	78(77.2%)	23(22.8%)	2.1(1.2–3.8)	0.015	2.1(1.2–3.9)	0.015
Low or normal	240(87.9%)	33(12.1%)	1.0		1.0	
**Education**						
Primary or below	120(81.1%)	28(18.9%)	1.3(0.8–2.1)	0.338	1.3(0.7–2.2)	0.391
University or above	84(79.2%)	22(20.8%)	1.5(0.8–2.5)	0.176	1.6(0.9–2.8)	0.098
Intermediate	339(84.5%)	62(15.5%)	1.0		1.0	
**Employment status**						
Working	380(83.9%)	73(16.1%)	1.1(0.6–1.8)	0.825	1.2(0.7–2.1)	0.581
Others	39(70.9%)	16(29.1%)	2.2(1.1–4.6)	0.035	2.5(1.2–5.5)	0.017
Retired/homemakers	124(84.4%)	23(15.6%)	1.0		1.0	
**Limited self-efficacy and competence**						
**BMI**						
Overweight	246(87.9%)	34(12.1%)	1.4(0.8–2.4)	0.266	1.7(0.9–3.1)	0.077
Obese	81(80.2%)	20(19.8%)	2.4(1.3–4.6)	0.007	2.8(1.4–5.5)	0.003
Low or normal	249(90.9%)	25(9.1%)	1.0		1.0	
**Age group**						
40–59	231(85.2%)	40(14.8%)	1.8(1.0–3.2)	0.044	1.8(1.0–3.3)	0.050
60–75	140(88.6%)	18(11.4%)	1.3(0.7–2.5)	0.471	1.3(0.5–3.0)	0.564
20–39	206(91.2%)	20(8.8%)	1.0		1.0	
**Education**						
Intermediate	361(90.0%)	40(10.0%)	0.82(0.45–1.50)	0.525	1.3(0.7–2.6)	0.443
University or above	85(80.2%)	21(19.8%)	1.8(0.9–3.6)	0.091	3.5(1.5–8.4)	0.004
Primary or below	130(88.4%)	17(11.6%)	1.0		1.0	
**Employment status**						
Others	47(85.5%)	8(14.5%)	1.4(0.6–3.1)	0.463	1.7(0.7–4.1)	0.223
Retired/homemakers	124(84.9%)	22(15.1%)	1.5(0.9–2.6)	0.129	1.5(0.7–3.1)	0.316
Working	405(89.4%)	48(10.6%)	1.0		1.0	
**Gender**						
Female	270(83.9%)	52(16.1%)	2.3(1.4–3.8)	0.001	2.6(1.5–4.5)	0.001
Male	306(92.2%)	26(7.8%)	1.0		1.0	
**Lack of perceived susceptibility, benefits and intentions**						
**BMI**						
Overweight	223(79.6%)	57(20.4%)	1.8(1.1–2.9)	0.012	1.9(1.2–3.0)	0.008
Obese	90(89.1%)	11(10.9%)	0.85(0.41–1.75)	0.652	1.0(0.5–2.1)	0.985
Low or normal	239(87.5%)	34(12.5%)	1.0		1.0	
**Age group**						
20–39	180(79.6%)	46(20.4%)	1.9(1.1–3.4)	0.028	1.9(1.0–3.7)	0.068
40–59	233(86.0%)	38(14.0%)	1.2(0.7–2.2)	0.523	1.2(0.6–2.2)	0.644
60–75	139(88.5%)	18(11.5%)	1.0		1.0	
**Education**						
Primary or below	127(86.4%)	20(13.6%)	1.0(0.6–1.7)	0.979	1.3(0.7–2.3)	0.461
University or above	80(75.5%)	26(24.5%)	2.1(1.2–3.5)	0.007	1.9(1.1–3.3)	0.016
Intermediate	346(86.3%)	55(13.7%)	1.0		1.0	
**Perceived lack of physical capability**						
**BMI**						
Overweight	212(75.7%)	68(24.3%)	1.9(1.2–3.0)	0.003	1.7(1.1–2.7)	0.017
Obese	69(68.3%)	32(31.7%)	2.7(1.6–4.7)	<0.001	2.4(1.4–4.2)	0.003
Low or normal	234(85.7%)	39(14.3%)	1.0		1.0	
**Age group**						
40–59	208(76.8%)	63(23.2%)	2.3(1.4–3.8)	0.001	1.9(1.2–3.3)	0.010
60–75	107(67.7%)	51(32.3%)	3.6(2.1–6.1)	<0.001	2.1(1.1–4.0)	0.026
20–39	200(88.5%)	26(11.5%)	1.0		1.0	
**Education**						
Primary or below	102(69.4%)	45(30.6%)	2.0(1.3–3.1)	0.002	1.0(0.6–1.7)	0.908
University or above	85(80.2%)	21(19.8%)	1.1(0.7–1.9)	0.680	1.4(0.8–2.5)	0.246
Intermediate	328(81.8%)	73(18.2%)	1.0		1.0	
**Employment status**						
Working	377(83.2%)	76(16.8%)	1.4(0.6–3.2)	0.445	1.4(0.6–3.3)	0.434
Retired/homemakers	90(61.6%)	56(38.4%)	4.3(1.8–10.0)	0.001	3.4(1.4–8.2)	0.008
Others	48(87.3%)	7(12.7%)	1.0		1.0	

Note: OR = 1.0 is the reference category.

#### Predictors of motivator factors in the physically inactive

Significant differences for some motivator factors were noted by sociodemographic characteristics and BMI. Intermediate level education was significantly associated with lower motivation as compared to university level education or above (adjusted OR 1.9, 95% CI 1.1–3.2, p = 0.018). For the motivator factor “external cues”, primary education or below was significantly associated with being low in this factor compared to those of intermediate education (adjusted OR 2.0, 95% CI 1.2–3.2, p = 0.004). Those low in the motivator factor of “significant others (family or friends)” were significantly more likely to be those with university level education or above compared to those with intermediate education (adjusted OR 4.5, 95% CI 1.9–10.7, p = 0.001). For “outcome expectations”, those aged 60–75 seemed more likely to have low motivations as compared to those aged 40–59, with borderline significance (adjusted OR 1.569, 95% CI 1.004–2.453, p = 0.048) (Table 3).

#### Predictors of barrier factors in the physically inactive

Significant differences for some barrier factors were noted by sociodemographic characteristics and BMI. For the “limited self-efficacy and competence” factor, those with university level education or above were significantly more likely to be high in this barrier factor as compared to primary level education or below (adjusted OR 3.5, 95% CI 1.5–8.4, p = 0.004). For the “lack of perceived susceptibility, benefits and intentions”, those with university level education or above were significantly more likely to be high in this barrier factor as compared to intermediate level education (adjusted OR 1.9, 95% CI 1.1–3.3, p = 0.016). The barrier factor of “perceived lack of physical capability” was significantly higher in those aged 40 to 59 and 60 to 75 compared to those aged 20 to 39 (adjusted OR 1.9, 95% CI 1.2–3.3, p = 0.010 and adjusted OR 2.1, 95% CI 1.1–4.0, p = 0.026, respectively) (Table 3).

### The smokers

#### Total scores of motivators and barriers in smokers

Those aged 60–75 were significantly more likely to have high barriers as compared to those aged 20–39 (adjusted OR 9.1, 95% CI 2.6–32.3, p = 0.001). Those with intermediate level education and university level education or above were significantly more likely to have high barriers as compared to those with primary level education or below (adjusted OR 5.0, 95% CI 1.2–20.6, p = 0.027 and adjusted OR 7.4, 95% CI 1.3–43.3, p = 0.025, respectively) (Table 4).

**Table 4:** Motivators and barriers among smokers: Overall score and factors.

**Motivators (total score)**						
**Variable**	**Motivated**	**Very Unmotivated**	**Unadjusted**		**Adjusted**	
			**OR (95% CI)**	**P-value**	**OR (95% CI)**	**P- value**
**BMI**						
Low or normal	75(75.0%)	25(25.0%)	1.8(0.9–3.6)	0.097	2.2(1.0–4.5)	0.040
Obese	26(78.8%)	7(21.2%)	1.4(0.5–3.7)	0.527	1.0(0.4–2.9)	0.928
Overweight	92(84.4%)	17(15.6%)	1.0		1.0	
**Physical activity levels**						
Didn’t meet recommendation	122(71.8%)	48(28.2%)	44.6(3.6–551.7)	0.003	50.4(4.0–628.7)	0.002
Meet recommendation	71(98.6%)	1(1.4%)	1.0		1.0	
**Motivator factors**						
**Variable**	**High score**	**Very low score**	**Unadjusted**		**Adjusted**	
			**OR (95% CI)**	**P-value**	**OR (95% CI)**	**P- value**
**Outcome expectations**						
**Employment status**						
Working	117(65.7%)	61(34.3%)	2.4(1.0–5.7)	0.041	2.4(1.0–5.7)	0.051
Others	17(77.3%)	5(22.7%)	1.4(0.4–5.1)	0.570	1.3(0.4–4.9)	0.653
Retired/homemakers	34(82.9%)	7(17.1%)	1.0		1.0	
**Physical activity levels**						
Didn’t meet recommendation	106(62.4%)	64(37.6%)	3.6(1.7–7.5)	0.001	3.6(1.7–7.5)	0.001
Meet recommendation	61(85.9%)	10(14.1%)	1.0		1.0	
**External cues**						
**Physical activity levels**						
Didn’t meet recommendation	115(67.6%)	55(32.4%)	4.1(1.8–9.4)	0.001	NA	NA
Meet recommendation	64(90.1%)	7(9.9%)	1.0			
**Significant others (family or friends)**						
**BMI**						
Low or normal	91(91.0%)	9(9.0%)	10.2(0.3–373.5)	0.206	12.3(0.3–463.1)	0.175
Overweight	106(97.2%)	3(2.8%)	2.9(0.1–118.7)	0.578	2.6(0.1–111.3)	0.613
Obese	33(100.0%)	0(0.0%)	1.0		1.0	
**Education**						
Primary or below	45(90.0%)	5(10.0%)	5.3(1.2–22.5)	0.024	6.4(1.5–28.2)	0.014
University or above	25(86.2%)	4(13.8%)	7.7(1.7–35.5)	0.009	9.6(2.0–47.1)	0.005
Intermediate	158(98.1%)	3(1.9%)	1.0		1.0	
**Barriers (total score)**						
**Variable**	**Low**	**Very high**	**Unadjusted**		**Adjusted**	
			**OR (95% CI)**	**P-value**	**OR (95% CI)**	**P- value**
**BMI**						
Low or normal	84(84.0%)	16(16.0%)	3.3(1.2–9.0)	0.018	3.7(1.3–10.7)	0.015
Obese	29(87.9%)	4(12.1%)	2.8(0.8–10.2)	0.125	3.1(0.8–12.3)	0.108
Overweight	103(94.5%)	6(5.5%)	1.0		1.0	
**Age group**						
40–59	91(89.2%)	11(10.8%)	2.3(0.7–7.3)	0.173	2.6(0.8–8.7)	0.114
60–75	44(80.0%)	11(20.0%)	4.8(1.5–15.9)	0.009	9.1(2.6–32.3)	0.001
20–39	80(95.2%)	4(4.8%)	1.0		1.0	
**Education**						
Intermediate	142(88.2%)	19(11.8%)	2.5(0.7–9.6)	0.178	5.0(1.2–20.6)	0.027
University or above	25(86.2%)	4(13.8%)	2.9(0.6–14.8)	0.209	7.4(1.3–43.3)	0.025
Primary or below	48(94.1%)	3(5.9%)	1.0		1.0	
**Barrier factors**						
**Variable**	**High score**	**Very low score**	**Unadjusted**		**Adjusted**	
			**OR (95% CI)**	**P-value**	**OR (95% CI)**	**P- value**
**External circumstances**						
**Age group**						
40–59	85(83.3%)	17(16.7%)	2.2(0.9–5.5)	0.105	2.0(0.8–5.1)	0.148
60–75	44(80.0%)	11(20.0%)	2.7(1.0–7.6)	0.051	2.5(0.9–7.0)	0.078
20–39	77(91.7%)	7(8.3%)	1.0		1.0	
**Physical activity levels**						
Didn’t meet recommendation	140(82.4%)	30(17.6%)	2.9(1.1–8)	0.036	2.7(1.0–7.4)	0.053
Meet recommendation	66(93.0%)	5(7.0%)	1.0		1.0	
**Limited self-efficacy and competence**						
**BMI**						
Low or normal	85(85.9%)	14(14.1%)	2.5(1.0–6.7)	0.057	2.7(1.0–7.5)	0.058
Obese	29(87.9%)	4(12.1%)	2.4(0.7–8.4)	0.182	1.8(0.5–7.0)	0.391
Overweight	102(93.6%)	7(6.4%)	1.0		1.0	
**Age group**						
40–59	87(85.3%)	15(14.7%)	4.0(1.2–13.6)	0.024	5.1(1.5–17.7)	0.010
60–75	48(87.3%)	7(12.7%)	3.5(0.9–13.3)	0.067	7.1(1.7–28.8)	0.006
20–39	81(96.4%)	3(3.6%)	1.0		1.0	
**Education**						
Intermediate or above	166(86.9%)	25(13.1%)	24.4(0.7–852.1)	0.078	51.7(1.4–1850.9)	0.031
Primary or below	50(100.0%)	0(0.0%)	1.0		1.0	
**Physical activity levels**						
Didn’t meet recommendation	147(86.5%)	23(13.5%)	3.6(1.0–12.7)	0.045	4.5(1.2–16.7)	0.025
Meet recommendation	68(95.8%)	3(4.2%)	1.0		1.0	
**Lack of perceived susceptibility, benefits and intentions**						
**Age group**						
40–59	89(87.3%)	13(12.7%)	1.7(0.6–4.6)	0.288	2.1(0.7–5.6)	0.163
60–75	44(80.0%)	11(20.0%)	3.0(1.1–8.5)	0.034	4.6(1.5–13.8)	0.007
20–39	78(91.8%)	7(8.2%)	1.0		1.0	
**Education**						
Intermediate	137(85.1%)	24(14.9%)	2.2(0.7–6.7)	0.178	4.4(1.3–14.6)	0.016
University or above	27(90.0%)	3(10.0%)	1.2(0.2–6.1)	0.845	2.3(0.4–12.6)	0.352
Primary or below	47(92.2%)	4(7.8%)	1.0		1.0	
**Physical activity levels**						
Didn’t meet recommendation	143(84.1%)	27(15.9%)	3.0(1.0–8.9)	0.043	3.3(1.1–9.9)	0.033
Meet recommendation	67(94.4%)	4(5.6%)	1.0		1.0	
**Perceived lack of physical capability**						
**Age group**						
40–59	82(81.2%)	19(18.8%)	4.2(1.4–12.3)	0.009	3.6(1.2–10.7)	0.022
60–75	41(74.5%)	14(25.5%)	6.2(2.0–19.3)	0.001	4.0(1.1–14.4)	0.034
20–39	80(95.2%)	4(4.8%)	1.0		1.0	
**Employment status**						
Working	154(86.5%)	24(13.5%)	1.3(0.3–5.6)	0.703	1.5(0.3–6.7)	0.604
Retired/homemakers	30(71.4%)	12(28.6%)	3.4(0.7–15.7)	0.117	2.9(0.6–14.9)	0.192
Others	19(90.5%)	2(9.5%)	1.0		1.0	
**Physical activity levels**						
Didn’t meet recommendation	135(79.4%)	35(20.6%)	5.5(1.7–17.8)	0.005	5.6(1.7–18.7)	0.005
Meet recommendation	68(95.8%)	3(4.2%)	1.0		1.0	

Note: OR = 1.0 is the reference category.

#### Predictors of motivator factors in smokers

For the factor of “significant others (family or friends)”, primary level education or below and university level education or above were significantly associated with being low in this factor as compared to intermediate level education (adjusted OR 6.4, 95% CI 1.5–28.2, p = 0.014 and adjusted OR 9.6, 95% CI 2.0–47.1, p = 0.005, respectively) (Table 4).

#### Predictors of barrier factors in smokers

For the barrier factor of “limited self-efficacy and competence”, smokers aged 40 to 59 and 60 to 75 were significantly more likely to be high in this factor compared to those aged 20 to 39 (adjusted OR 5.0, 95% CI 1.4–17.6, p = 0.012 and adjusted OR 8.0, 95% CI 1.9–33.4, p = 0.004, respectively). Intermediate education or above was significantly associated with being high in this barrier factor as compared to primary level education or below (adjusted OR 51.7, 95% CI 1.4–1850.9, p = 0.031). For the barrier factor of “lack of perceived susceptibility, benefits and intentions”, those aged 60 to 75 were significantly more likely to be high in this factor compared to those aged 20 to 39 (adjusted OR 4.6, 95% CI 1.5–13.8, p = 0.007). Those with intermediate level education were significantly more likely to be high in this barrier factor as compared to primary level education or below (adjusted OR 4.4, 95% CI 1.3–14.6, p = 0.016). The barrier factor of “perceived lack of physical capability” was significantly higher in those aged 40 to 59 and those aged 60 to 75 as compared to those aged 20 to 39 (adjusted OR 3.6, 95% CI 1.2–10.7, p = 0.022 and adjusted OR 4.0, 95% CI 1.1–14.4, p = 0.034, respectively) (Table 4).

### The obese

#### Total scores of motivators and barriers in the obese

Among the obese, those aged 60–75 were significantly more likely to have low motivations as compared to those aged 40–59 (adjusted OR 4.4, 95% CI 1.1–17.2, p = 0.035, Table 5). Those with primary level education or below were significantly more likely to have low motivations as compared to those with intermediate level education or below (adjusted OR 4.0, 95% CI 1.2–14.1, p = 0.028). There was no significant association between sociodemographic variables and barriers (Table 5).

**Table 5:** Motivators and barriers among the obese: Overall score and factors.

**Motivators (total score)**						
**Variable**	**Motivated**	**Very Unmotivated**	**Unadjusted**		**Adjusted**	
			**OR (95% CI)**	**P-value**	**OR (95% CI)**	**P- value**
**Age group**						
20–39	28(87.5%)	4(12.5%)	1.6(0.4–5.9)	0.484	4.3(0.9–21.0)	0.070
60–75	29(70.7%)	12(29.3%)	4.2(1.4–12.2)	0.009	4.4(1.1–17.2)	0.035
40–59	59(90.8%)	6(9.2%)	1.0		1.0	
**Education**						
Primary or below	35(72.9%)	13(27.1%)	3.8(1.4–10.0)	0.008	4.0(1.2–14.1)	0.028
University or above	6(75.0%)	2(25.0%)	3.3(0.6–19.9)	0.185	6.7(0.9–51.9)	0.068
Intermediate	76(90.5%)	8(9.5%)	1.0		1.0	
**Employment status**						
Others	10(76.9%)	3(23.1%)	2.6(0.6–10.9)	0.195	2.2(0.5–10.3)	0.324
Retired/homemakers	34(77.3%)	10(22.7%)	2.4(0.9–6.4)	0.077	1.3(0.3–5.2)	0.685
Working	72(88.9%)	9(11.1%)	1.0		1.0	
**Physical activity levels**						
Low	80(79.2%)	21(20.8%)	4.8(1.1–21.8)	0.043	5.5(1.1–26.5)	0.035
Meet recommendation	36(94.7%)	2(5.3%)	1.0		1.0	
**Motivator factors**						
**Variable**	**High score**	**Very low score**	**Unadjusted**		**Adjusted**	
			**OR (95% CI)**	**P-value**	**OR (95% CI)**	**P- value**
**Outcome expectations**						
**Race**						
Chinese	53(66.3%)	27(33.8%)	1.9(0.8–4.5)	0.170	1.8(0.7–4.6)	0.197
Indian	13(72.2%)	5(27.8%)	1.4(0.4–5.0)	0.591	1.3(0.4–4.9)	0.672
Malay	31(77.5%)	9(22.5%)	1.0		1.0	
**Employment status**						
Working	54(65.9%)	28(34.1%)	1.9(0.8–4.5)	0.140	1.7(0.7–4.2)	0.219
Others	8(66.7%)	4(33.3%)	1.9(0.5–7.5)	0.335	1.9(0.5–8.1)	0.372
Retired/homemakers	35(79.5%)	9(20.5%)	1.0		1.0	
**Physical activity levels**						
Didn’t meet recommendation	64(63.4%)	37(36.6%)	3.9(1.4–10.9)	0.010	3.4(1.2–9.7)	0.023
Meet recommendation	33(86.8%)	5(13.2%)	1.0		1.0	
**External cues**						
**Age group**						
20–39	28(87.5%)	4(12.5%)	0.53(0.15–1.83)	0.314	1.4(0.3–6.0)	0.634
60–75	25(61.0%)	16(39.0%)	2.6(1.1–6.1)	0.033	4.1(1.4–11.4)	0.008
40–59	52(80.0%)	13(20.0%)	1.0		1.0	
**Education**						
Primary or below	31(64.6%)	17(35.4%)	3.2(1.4–7.6)	0.007	3.2(1.1–9.0)	0.028
University or above	3(37.5%)	5(62.5%)	8.6(1.8–40.7)	0.007	13.8(2.5–77.8)	0.003
Intermediate	71(85.5%)	12(14.5%)	1.0		1.0	
**Gender**						
Male	40(70.2%)	17(29.8%)	1.7(0.8–3.8)	0.173	2.7(1.0–6.9)	0.043
Female	65(80.2%)	16(19.8%)	1.0		1.0	
**Physical activity levels**						
Didn’t meet recommendation	72(71.3%)	29(28.7%)	3.6(1.2–11.4)	0.026	3.6(1.0–12.8)	0.043
Meet recommendation	34(89.5%)	4(10.5%)	1.0		1.0	
**Significant others (family or friends)**						
**Age group**						
20–39	28(87.5%)	4(12.5%)	3.6(0.7–18.2)	0.120	8.3(0.8–80.3)	0.069
60–75	38(90.5%)	4(9.5%)	2.5(0.5–12.8)	0.263	2.9(0.4–23.2)	0.308
40–59	63(95.5%)	3(4.5%)	1.0		1.0	
**Education**						
Primary or below	43(89.6%)	5(10.4%)	2.0(0.5–8.0)	0.327	3.2(0.4–23.8)	0.246
University or above	6(75.0%)	2(25.0%)	6.5(1.0–43.1)	0.054	35.0(2.1–587.2)	0.014
Intermediate	79(95.2%)	4(4.8%)	1.0		1.0	
**Gender**						
Female	72(87.8%)	10(12.2%)	8.2(1.0–70.4)	0.056	5.2(0.5–51.5)	0.157
Male	56(98.2%)	1(1.8%)	1.0		1.0	
**Currently smoking**						
No	96(90.6%)	10(9.4%)	11.4(0.3–412.8)	0.185	14.9(0.3–822.1)	0.187
Yes	33(100.0%)	0(0.0%)	1.0		1.0	
**Barriers (total score)**						
**Variable**	**Low**	**Very high**	**Unadjusted**		**Adjusted**	
			**OR (95% CI)**	**P-value**	**OR (95% CI)**	**P- value**
**Age group**						
20–39	28(87.5%)	4(12.5%)	1.3(0.3–4.9)	0.730	1.3(0.3–5.0)	0.751
60–75	28(66.7%)	14(33.3%)	4.7(1.7–13.3)	0.004	3.1(1–9.9.0)	0.056
40–59	59(90.8%)	6(9.2%)	1.0		1.0	
**Employment status**						
Others	11(84.6%)	2(15.4%)	1.2(0.2–7.5)	0.837	1.0(0.2–6.8)	0.960
Retired/homemakers	30(68.2%)	14(31.8%)	4.2(1.6–10.9)	0.003	2.2(0.7–7.0)	0.167
Working	73(90.1%)	8(9.9%)	1.0		1.0	
**Gender**						
Female	64(79.0%)	17(21.0%)	2.0(0.8–5.4)	0.147	1.6(0.6–4.6)	0.387
Male	51(87.9%)	7(12.1%)	1.0		1.0	
**Barrier factors**						
**Variable**	**High score**	**Very low score**	**Unadjusted**		**Adjusted**	
			**OR (95% CI)**	**P-value**	**OR (95% CI)**	**P- value**
**External circumstances**						
**Age group**						
40–59	54(83.1%)	11(16.9%)	1.5(0.4–5.3)	0.510	1.4(0.4–5.1)	0.569
60–75	28(66.7%)	14(33.3%)	3.7(1.1–12.9)	0.039	2.4(0.6–9.6)	0.208
20–39	28(87.5%)	4(12.5%)	1.0		1.0	
**Employment status**						
Working	69(85.2%)	12(14.8%)	1.7(0.2–11.6)	0.613	1.8(0.3–13.1)	0.552
Retired/homemakers	29(65.9%)	15(34.1%)	5.1(0.7–35.5)	0.103	3.6(0.5–27.8)	0.211
Others	12(92.3%)	1(7.7%)	1.0		1.0	
**Currently smoking**						
No	81(76.4%)	25(23.6%)	2.1(0.7–6.6)	0.187	1.4(0.4–4.6)	0.624
Yes	29(87.9%)	4(12.1%)	1.0		1.0	
**Limited self-efficacy and competence**						
**Age group**						
40–59	55(84.6%)	10(15.4%)	2.9(0.6–14.7)	0.200	2.9(0.4–19.6)	0.276
60–75	29(70.7%)	12(29.3%)	6.8(1.4–34.5)	0.020	6.7(0.9–50.7)	0.065
20–39	30(93.8%)	2(6.3%)	1.0		1.0	
**Education**						
Intermediate	74(89.2%)	9(10.8%)	0.50(0.19–1.32)	0.162	1.5(0.5–4.9)	0.506
University or above	3(37.5%)	5(62.5%)	7.9(1.5–41.2)	0.014	64.7(7.2–578.9)	<0.001
Primary or below	38(79.2%)	10(20.8%)	1.0		1.0	
**Employment status**						
Others	12(92.3%)	1(7.7%)	0.66(0.07–6.09)	0.715	1.6(0.1–18.9)	0.706
Retired/homemakers	30(66.7%)	15(33.3%)	4.2(1.6–10.8)	0.003	4.9(1.2–20.0)	0.026
Working	73(89.0%)	9(11.0%)	1.0		1.0	
**Gender**						
Female	61(75.3%)	20(24.7%)	4.7(1.5–14.7)	0.009	5.0(1.3–19.6)	0.022
Male	54(93.1%)	4(6.9%)	1.0		1.0	
**Perceived lack of physical capability**						
**Age group**						
40–59	48(73.8%)	17(26.2%)	2.4(0.7–7.6)	0.150	1.6(0.5–5.7)	0.450
60–75	22(52.4%)	20(47.6%)	6.2(1.9–20.7)	0.003	3.1(0.8–12.7)	0.111
20–39	28(87.5%)	4(12.5%)	1.0		1.0	
**Education**						
Primary or below	27(56.3%)	21(43.8%)	3.2(1.4–6.9)	0.004	1.9(0.8–4.7)	0.163
University or above	4(50.0%)	4(50.0%)	4.2(0.9–18.9)	0.063	5.7(1.2–28.4)	0.033
Intermediate	67(80.7%)	16(19.3%)	1.0		1.0	
**Employment status**						
Working	64(78.0%)	18(22.0%)	2.7(0.4–18.2)	0.318	2.7(0.4–19.8)	0.316
Retired/homemakers	22(50.0%)	22(50.0%)	9.2(1.3–63.9)	0.025	5.6(0.7–41.9)	0.096
Others	12(92.3%)	1(7.7%)	1.0		1.0	
**Currently smoking**						
No	70(66.0%)	36(34.0%)	2.6(1.0–7.2)	0.062	1.7(0.6–5.4)	0.343
Yes	28(84.8%)	5(15.2%)	1.0		1.0	

Note: OR = 1.0 is the reference category.

#### Predictors of motivator factors in the obese

For “external cues”, those aged 60 to 75 as compared to those aged 40 to 59 were significantly more likely to be low in this motivation factor (unadjusted OR 4.1, 95% CI 1.4–11.4, p = 0.008). For “significant others (family or friends)”, those with university level education or above were significantly more likely to be low in this factor as compared to intermediate level education (adjusted OR 35.0, 95% CI 2.1–587.2, p = 0.014) (Table 5).

#### Predictors of barrier factors in the obese

Those with university level education or above were significantly more likely to be high in the barrier factor of “lack of perceived susceptibility, benefits and intentions” (adjusted OR 64.7, 95% CI 7.2–578.9, p<0.001) compared to those who were of primary level education or below. The retired or homemakers were significantly more likely to be high in the barrier factor of “limited self-efficacy and competence” (adjusted OR 4.9, 95% CI 1.2–20.0, p = 0.026) compared to those who were in workforce. Those of university level education or above were significantly more likely than those of intermediate level education to be high in the barrier factor of “perceived lack of physical capability” (adjusted OR 5.7, 95% CI 1.2–28.4, p = 0.033) (Table 5). Overall, race (Chinese, Malay or Indian) was not a significant factor determining the likelihood of having motivators or barriers to heart healthy behaviors.

## Discussion

This study examined the patterns of motivators and barriers influencing heart health behaviors in adult population segments with three behavior-modifiable risk factors (physical inactivity, smoking and obesity). Overall motivations were more likely to be low in those with lower education (for the physically inactive and the obese) whereas overall barriers were more likely to be high in those with higher education (for the physically inactive and the smokers). This finding of overall higher perceived barriers in the well-educated was also seen in Japan [12].

We found that individuals with lower education were more likely to have low motivation factor of “outcome expectations” (for the physically inactive). This indicates that people with lower education tended not to prioritize long-term health benefits of heart health behavior. This finding is in line with studies that people with lower socio-economic status in terms of income and education were less likely to prioritize long-term health benefits [24, 25]. Another finding is that individuals with lower education had low motivation factor of “external cues” which represent public health campaigns or advice from healthcare professionals (for the physically inactive). This affirms existing evidence of generally poor receptivity of health promotion efforts [26] among people of low education. However, in our study, people with lower education were found to be motivated by “significant others (family or friends)” (for the physically inactive and the obese). As with the studies that found the beneficial effects of social influence in improving uptake of physical activity, it may be useful to engage family and friends in interventions to increase motivation in the lower education group. Interventions for this group should also consider shifting individual’s priorities to health benefits and guiding them to see the immediate relevance of health benefits to them.

This study showed that a key barrier to heart health behaviors in individuals with higher education is “limited self-efficacy and competence” (for the physically inactive and the obese). A possible explanation could be that the highly educated do place importance on the benefits of health behaviors, and hence more have tried to change, albeit lack of success. This finding is different from studies in Saudi Arabia [27] and Brazil [25]. Our findings also showed that the well-educated perceived had low perceived susceptibility to the risk of heart disease (for the physically inactive) despite their poor heart health behaviors and did not anticipate the benefits of behavior change (for the obese). Strategies to improve self-efficacy and alter personal beliefs should consider developing role models and evidence-based information to allow the well-educated to better visualize the link between behavior modifiable risk factors and CVDs.

An interesting finding in the well-educated (among the obese, smokers and the physically inactive) was that they were less likely to have the motivation factor of “significant others (family or friends)” compared to those with intermediate levels of education. This finding is different from that of a study in Brazil where those with more schooling perceived more social support for behavioral change [25]. There are various reasons which may account for the differences. Firstly, unlike the present study, the Brazilian study did not specifically focus on population segments with poor health behaviors. In addition, various social and cultural contexts may explain the differences. Taken together, it becomes evident that groups with very low levels of education and groups with very high levels of education are less effectively motivated by significant others such as family and friends in comparison to those of intermediate level of education. This suggests that further research can focus on investigating the best ways to help the well-educated group gain social motivation, as social influence has been shown to have beneficial effects on health behaviors when applied positively [28, 29].

Lastly, an important finding was the oldest age group of 60 to 75 years was more likely to have overall low motivations (among the obese) and high barriers (among smokers) compared to other age groups. This finding indicates potential challenges in altering health behaviors for this age group. In terms of underlying motivator and barrier factors, the oldest age group in this study were more likely to be low in motivation factors of “external cues” (among the obese) and high in barrier factors of “lack of physical capability” (among the physically inactive and smokers), “limited self-efficacy and competence” and “lack of perceived susceptibility, benefits and intentions” (among the smokers). This is in contrast with a study in Japan that the elderly had overall better motivations compared to their younger counterparts [12]. These age-specific findings illuminate urgent intervention needs for the older adults with poor cardiovascular health behavior; as shown in this study, they may be particularly difficult to change as they have a myriad of barriers and lack motivators.

The strengths of this study included a large population-based sample of adult segments with behavior-modifiable risk factors, allowing for an understanding of the problems specifically affecting this group.

This study has a few limitations. The self-reported nature of the data means that findings may need to be treated with some caution due to recall or social desirability bias. Another limitation is related to the survey which limited options of motivators and barriers. It remains uncertain whether there would be more factors enabling or hindering heart healthy behaviors in this population.

In conclusion, this study found variability in the patterns of motivators and barriers affecting heart health behaviors in different population segments with behavior-modifiable risk factors. Those with lower education in general felt less motivated to make behavior changes for heart health while the well-educated were not fully convinced of the effectiveness of their actions in improving heart health. The well-educated also more keenly felt their past failures in behavioral change and lacked confidence in their ability to succeed. People aged 60 and above with poor behaviors were especially resistant to change and will likely need sustained efforts to change their attitudes. The patterns seen in the Asian population segments with behavioral risks will inform the design of future intervention and communication strategies addressing specific motivators and barriers.

## Supporting information

S1 Data (DTA)
