# Supplementary material for: Patterns of motivators and barriers to heart health behaviors among adults with behavior-modifiable cardiovascular risk factors: A population-based survey in Singapore
Source: PLoS One. 2022 Jan 20;17(1):e0262752. doi: 10.1371/journal.pone.0262752 (PMC8775312; doi:10.1371/journal.pone.0262752)
Supplement: Attachment — Submitted filename: Response to Reviewers (2).docx [file pone.0262752.s002.docx]

Dr Jagan Kumar Baskaradoss

Academic Editor

PLOS ONE

Dear Dr Baskaradoss,

Please find attached a revised version of the manuscript entitled, Patterns of motivators and barriers to heart health behaviors among adults with behavior-modifiable cardiovascular risk factors: a population-based survey in Singapore. We are grateful to the reviewer for the valuable suggestions.

We hope that the revisions in the manuscript and our accompanying responses will be sufficient to warrant publication in PLOS ONE.

We shall look forward to your positive response. Thank you very much.

**Point-by-point response**

1. Introduction: “identify motivators and barriers to uptake of heart health behavior ” – Meaning is unclear – rephrase

**Response**: Thanks for this comment. We have rephrased as “conducted a population survey to examine the factors that motivate and hinder uptake of heart health behavior in our Asian population. These motivators and barriers have previously been surveyed in the Western population (ref).”

2. For the subheading “Behavior-modifiable Cardiovascular Risk Factors: Physical inactivity, Smoking and Obesity” Add reference for each of the variables assessed (eg: MET, Smoking, BMI etc)

**Response**: References have been added for each of the variables assessed.

3. What was the distribution of the data for motivators are barriers – Why was mean score taken to dichotomize

**Response**: The distribution of the data for barriers was normal and the distribution of the data for motivators was slightly skewed. Therefore, we feel that it would be reasonable to use mean-1sd as a cut off to dichotomise.

4. Data was not provided as a supplementary file – please provide this

**Response**: We have now provided the data as a supplementary file.

5. Nationality of respondents should be included to Table 1

**Response**: All participants were Singaporeans or Permanent residents of Singapore as shown in the sample sub-section under Methods. In order to improve clarity, this has been stated again under Results in the sociodemographics sub-section.

6. Results: For- “outcome expectations”, those aged 60-75 were more likely to have low motivations as compared to those aged 40-59 (adjusted OR 1.6, 95% CI 1.0-2.5, p=0.048).” This is not statistically significant (OR included value 1). Revise the result accordingly.

**Response**: Thanks for this comment. We have amended the result to show 3 decimal places and to state that it is only borderline significant: “For “outcome expectations”, those aged 60-75 seemed more likely to have low motivations as compared to those aged 40-59, with borderline significance (adjusted OR 1.569, 95% CI 1.004-2.453, p=0.048).” We have also removed the outcome expectations from the discussion section given the marginally significant result.
